# Characteristics of person-centred practice in the stroke patient trajectory: a scoping review

**DOI:** 10.1186/s12883-025-04472-2

**Published:** 2025-12-01

**Authors:** Asma Sabri, Karina Aase, Sissel Iren Eikeland Husebø

**Affiliations:** SHARE – Centre for Resilience in Healthcare, Department of Quality and Health Technology, Faculty of Health Sciences, University of Stavanger, Stavanger, Norway

**Keywords:** Person-centred, Stroke, Review, Patient trajectory

## Abstract

**Background:**

Person-centred practice (PCP) is gaining increasing attention in healthcare practice and research. However, further knowledge is needed to understand PCP in stroke care. The aim of this study was to explore the characteristics of PCP in the stroke patient trajectory.

**Methods:**

The review was conducted using the JBI methodology for scoping reviews. Searches were conducted in MEDLINE, CINAHL, Embase, Cochrane Library, Scopus, PsycINFO, and Web of Science, along with grey literature sources. Two reviewers screened studies for inclusion. Data were extracted using a pilot-tested tool and analysed using content analysis for qualitative data and descriptive statistics for quantitative data.

**Results:**

Ninety-six studies from diverse healthcare contexts and countries were included. The results showed significant variability in the implementation of PCP in the stroke patient trajectory. Key components of PCP that dominate the literature include shared decision-making, holistic care, and information provision. Several categories of facilitators and barriers emerged from the data, including patient participation and interprofessional practice. Recommendations for practice emphasised the need for training healthcare professional in PCP principles and the development of tools to support shared decision-making. Research gaps include PCP gaps, theoretical, methodological, and implementation gaps, as well as tools and digital health gaps.

**Conclusions:**

This scoping review highlights the variability and complexity of implementing PCP in stroke care, emphasising the need for further research and development across the stroke patient trajectory.

**Supplementary Information:**

The online version contains supplementary material available at 10.1186/s12883-025-04472-2.

## Background

Stroke is one of the leading causes of death and disability in the world [1], imposing a burden on individuals and healthcare systems [2, 3]. This review focuses on the characteristics of person-centred practice (PCP) in the early phases of stroke.

Understanding the importance of person-centred approaches in stroke care requires recognising stroke heterogeneity in aetiologies, symptoms, recovery, and long-term outcomes. Each patient with stroke is unique due to differences in lesion localisation and size, as well as individual characteristics. The impact of stroke goes beyond physical impairments, affecting cognitive, psychological, and social aspects of patients’ lives, with 25% of patients being affected for a lifetime [4]. Research has demonstrated that the non-physical consequences of stroke—including emotional and psychological impacts, and social isolation—can present greater long-term challenges than physical limitations themselves [5].

Stroke trajectories and care processes vary considerably from patient to patient, and across regions and healthcare systems. This variety is due to multiple factors, including stroke heterogeneity and severity, access to care, and differences in healthcare infrastructures and policies [6]. This review focuses on the stroke patient trajectory defined as the first three months following stroke onset, divided into two phases: (1) the acute phase, defined as the first week following stroke onset, with the first 24 h defined as the hyperacute phase, and (2) the follow-up phase also referred to as the subacute phase, which continues until three months post-stroke, including rehabilitation and most commonly transition to home [7].

Although the long-term consequences of stroke are well documented, this review focuses on the early phases of stroke, as it is a critical period for initiating PCP, constituting a foundation for long-term outcomes. The early phases represent a critical window for recovery, and care coordination, during which the trajectory is most dynamic.

In the acute stroke phase, treatment effectiveness is time-dependent and patient conditions are usually critical [8]. The need to focus on medical expertise and patient stabilisation in the hyperacute phase might create a practical barrier to comprehensive patient engagement. However, assessment of patient needs is an integral part of acute care. Furthermore, patients and caregivers do not consider the lack of comprehensive engagement in the emergency department as negative, but rather the lack of information and communication [9, 10].

Stroke rehabilitation, which usually starts in the acute phase and spans the follow-up phase, places significant importance on shared goal setting, where patients identify meaningful rehabilitation objectives to improve engagement and satisfaction [11]. Other important elements include patient and caregiver education, psychological support, and active informal caregiver involvement [12]. Even so, rehabilitation services often prioritise regaining function and, to a lesser extent, enabling social participation and regaining meaningful roles and responsibilities [13].

Stroke care involves multiple care transitions. The transition from hospital to home is a critical juncture that typically occurs within the first few days to weeks after admission and can place significant burden on patients and their caregivers. This transition creates challenges such as lack of continuity and patient and caregiver strain, particularly given the decreasing length of hospital stays in many healthcare systems [14, 15]. However, PCP can improve the discharge process by actively involving patients in planning their care [16]. Patient participation usually evolves throughout recovery and patients who receive more information starting from an earlier point and having a follow-up plan experience a more satisfying homecoming [17].

PCP has gained attention as a fundamental component of providing high-quality healthcare [18]. While often used interchangeably with person-centred care, PCP refers to a broader framework that emphasises building and fostering a healthful relationship that supports shared decision-making, collaborative relationships, transformational leadership, and innovative practices [19]. Research demonstrates that PCP leads to positive outcomes in several healthcare contexts [20–22], such as improved satisfaction among patients, informal caregivers, and healthcare professionals, decreased hospitalisations, equal access to care, and potential cost effectiveness [23, 24]. For stroke patients, there has also been growing recognition of the impact of individualised approaches to neurorehabilitation on neuroplasticity and recovery [25, 26].

This review uses the PCP framework developed by McCormack and McCance, consisting of five interconnected layers: (1) prerequisites focusing on staff characteristics, (2) the care environment capturing the context in which services are delivered, (3) person-centred processes emphasising the practices that operationalise PCP such as shared decision-making and engaging authentically, and (4) outcomes representing the expected results. All elements operate within a broader (5) macro context of organisational systems, strategic, and policy frameworks [27].

Most studies on PCP that are context-specific and/or patient-group-specific have focused on chronic contexts, elderly persons, and patients with dementia [28]. There is a lack of a comprehensive overview of the literature on PCP in the context of stroke care [12, 29], and usually the focus is on one or a few aspects of PCP. This review provides stroke-specific knowledge and benefits from gathering data from the literature on PCP and its components in the stroke patient trajectory.

The aim of this scoping review is therefore to explore the characteristics of PCP in the stroke patient trajectory.

## Methods

A scoping review is well-suited for exploring the characteristics of PCP in the stroke patient trajectory due to the broad scope of the topic [30]. With the inclusion of diverse study designs, contexts, and populations, this approach allows for mapping the existing literature as well as identifying research gaps. This review was conducted using the Joanna Briggs Institute (JBI) methodology for scoping reviews [31, 32], involving nine steps: (1) defining and aligning objectives/questions, (2) developing and aligning inclusion criteria, (3) describing the approach, (4) searching the evidence, (5) selecting the evidence, (6) extracting the evidence, (7) analysing the evidence, (8) presenting the results, and (9) summarising the evidence in relation to the review’s purpose. As a reporting guideline, the Preferred Reporting Items for Systematic Reviews and Meta-Analyses extension for Scoping Reviews (PRISMA-ScR) was used [33]. More details of the methodology are provided in the review protocol published on the Open Science Framework [34].

### Identifying the research questions

To explore the characteristics of PCP in the stroke patient trajectory, the following research questions were constructed:

1. What are the demographic characteristics of the literature published on PCP in the stroke patient trajectory?
2. How is PCP in the stroke patient trajectory operationalised according to the PCP framework [27]?
3. What are the facilitators and barriers to PCP in the stroke patient trajectory?
4. What are the recommendations to improve PCP in the stroke patient trajectory?
5. What are the research gaps in PCP in the stroke patient trajectory, and how have they evolved over time?

Together, these questions progress from mapping the field (RQ1-2), identifying various facilitators and barriers (RQ3), summarising recommendations for practice (RQ4), and guiding future research by identifying research gaps (RQ5). This structure provides a comprehensive understanding of PCP in the stroke patient trajectory, supporting both theoretical development and practical implementation.

### Inclusion criteria

The Population, Concept, and Context (PCC) framework [31] was used to define the scope and identify relevant research literature (Table 1).

**Table 1:** Inclusion and exclusion criteria for selected articles

Criteria	Inclusion	Exclusion
Population	patients with stroke, informal caregivers, healthcare professionals involved in the stroke patient trajectory.	Studies not exclusively focused on stroke and studies including Transient Ischemic Attack (TIA), severe, or childhood stroke.
Concept	Person-centred, patient involvement, decision-making, family-centred.	Studies not addressing PCP or one of its components.
Context	The stroke patient trajectory, pre-hospital, in-hospital, post-hospital, acute care, subacute care, rehabilitation.	Studies outside the stroke patient trajectory and studies focused on primary or secondary prevention.
Study design	Empirical research studies	Non-empirical studies, protocols, and articles on psychometric testing
Language	English	Non-English

*Articles on severe stroke were excluded, as they primarily focused on palliative care and for practical reasons to reduce the number of full text articles

### Search strategy

As proposed in the JBI manual for scoping reviews [31], a three-step search strategy was conducted in January 2024. First, a preliminary search was performed by the first author using the PCP synonyms in the Databases MEDLINE and EMBASE to determine the keywords and index terms used in research on the topic. Second, in collaboration with an experienced librarian in healthcare research, the keywords and index terms used interchangeably in the literature to describe PCP were identified and used to develop a comprehensive search strategy for several databases. The search was then conducted in MEDLINE, CINAHL, Embase, Cochrane Library, Scopus, PsycINFO and Web of Science to identify relevant full-text articles. Additional searches were conducted in Google Scholar, Google, and ProQuest Dissertations & Theses Global (ProQuest) to identify more relevant studies and grey literature. The spelling of ‘centered’ versus ‘centred’ was considered by running each term (e.g., person-centered and person-centred). Third, the reference lists of relevant included studies were screened to identify further studies. The detailed search strategy for CINAHL is presented in Appendix I.

### Study selection process

Following the search, all identified citations were collated and uploaded into EndNote, and duplicates were removed. Two reviewers (AS, SEH) independently screened a sample of 30 abstracts for a pilot test in Rayyan [35], a web-based tool for systematic reviews, to ensure consistency in applying the eligibility criteria. Following this pilot test, titles and abstracts were screened in Rayyan with blinding enabled for two reviewers (AS, SEH) for assessment against the eligibility criteria. Potentially relevant sources were retrieved in full. The full text of selected citations was assessed in detail against the eligibility criteria by two reviewers (AS, SEH). In the screening process, the first reviewer screened all articles, while the second reviewer screened a randomly selected 10% of articles. Reasons for the exclusion of sources of evidence in the full text stage were recorded and reported. Disagreements between the reviewers at each stage of the selection process were resolved through discussion and consensus, or by involving a third reviewer (KA). The results of the search and the inclusion process were reported in full and presented in a PRISMA-ScR flow diagram.

During the study selection process, the PCP definition and framework were used to decide on inclusion of articles ensuring that the review covered all layers of the framework in relation to person-centredness. As a result, studies addressing for example leadership or professional practice without a clear connection to person-centredness were excluded. This targeted strategy helped maintain the feasibility of the review while acknowledging the breadth of the framework.

### Data extraction and analysis

Data were extracted from relevant articles using a data extraction tool developed by the three authors (AS, KA, SEH) based on the review questions and following the JBI guidelines [36]. Using a sample of 10% of the included articles, the data extraction tool (Appendix II) was piloted and assessed for completeness and applicability. It was then modified to ensure all necessary data to address the research questions were obtained. The characteristics of included studies were directly extracted from articles to address research question 1. For research question 2, a deductive approach was applied by analysing each article through the lens of the PCP framework by the three authors (AS, KA, SEH). The concepts and definitions from the framework were mapped in a reference document, which guided the data extraction and analysis. For research questions 3–5, an inductive approach was applied by two authors (AS, KA) using the three main phases of the content analysis process [37]. In the preparation phase, the extracted data were read several times for familiarisation. During the organisation phase, open coding was applied using NVivo 15 [38]. In the abstraction phase, subcategories were named using content words and grouped into categories allowing for the development of concepts covering facilitators and barriers, recommendations for practice, and research gaps. Results were reported using tables, graphs, and texts.

## Results

### Literature search

**Fig. 1:**
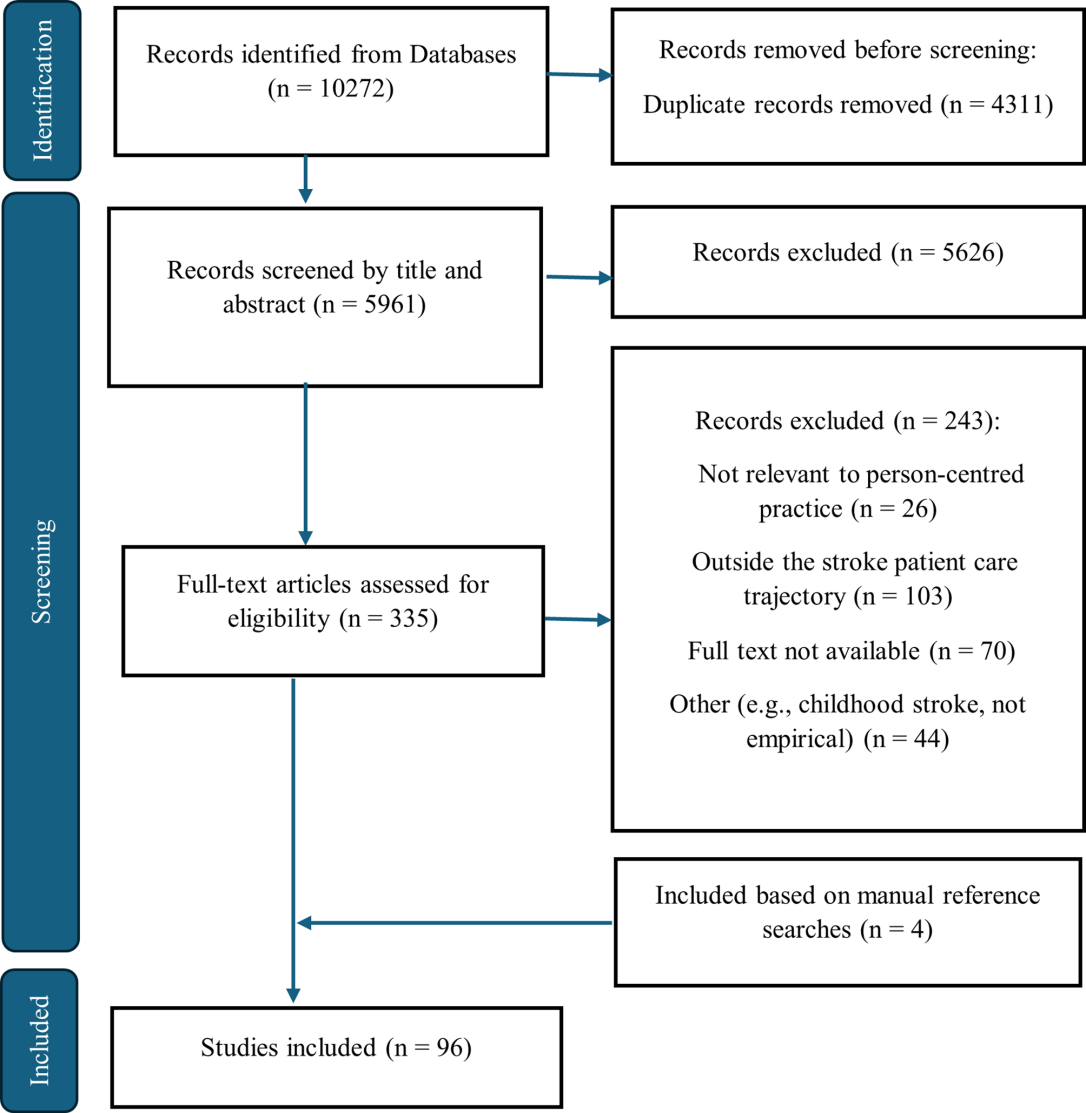
PRISMA flow diagram of the study selection process and outcomes

Our database search yielded 10,272 records in the initial search. After removing duplicates (*n* = 4311), 5961 articles remained for initial screening. The initial screening was conducted using titles and abstracts and resulted in the exclusion of 5626 articles. The full texts of the remaining articles (*n* = 335) were screened, and 243 articles were excluded for various reasons based on the exclusion criteria, with lack of PCP focus and not being within the stroke patient trajectory as the most frequent reasons. Further searches in grey literature, as well as reference lists of the included articles, resulted in four additional articles. In total, a sample of 96 studies was included in this review (Fig. 1).

### Characteristics of included studies

The results showed an increase in publications over the past three decades, with 70.5% of publications in the past eleven years (2013–2023) (Table 2). Research activity was restricted to Europe, North America, and the Asia-Pacific region, with Europe dominating (60%). Across regions, most article authors (65.7%) were from Australia, Sweden, the UK, the USA, and the Netherlands. Various study designs were used, with qualitative methods representing the majority (66.7%), while quantitative methods, mixed methods, and reviews were equally represented. The study population included patients in 31.3% of the studies, healthcare professionals (HCPs) in 28.1%, and informal caregivers in 6%, with most studies (34.7%) involving a mix of the three population groups. The total number of participants across all included studies was 11 384, in which 50% were patients, 26.4% caregivers, and 20.3% HCPs. There was considerable variation among studies, ranging from a single case with one participant to a review including 2 900 participants. Of the included studies, 43.2% covered the follow-up phase, 22% covered the acute phase, and 34.4% covered both. Within the follow-up phase, inpatient rehabilitation was predominant (54.1%), while transitional care and outpatient rehabilitation accounted for 10.8% and 1.4%, respectively.

**Table 2:** Characteristics of the literature on person-centred practice in the stroke patient trajectory

**Time periods**	**N**	**%**	
1993–2002 (10years)	7	7.3	
2003–2012 (10 years)	21	21.9	
2013–2023 (11 years)	68	70.8	
Total	96	100%	
Region	Countries (Count)	Region Total	%
Europe	Austria (2), Denmark (6), Finland (1), France (1), Germany (5), Ireland (2), the Netherlands (10), Norway (5), Sweden (15), Switzerland (2), UK (14)	63	60%
North America	Canada (3), USA (12)	15	14.3%
Asia–Pacific	Australia (18), China (1), Hong Kong (2), Japan (1), Malaysia (1), New Zealand (3), Singapore (1)	27	25.7%
Total		105*	100%
		N	%
Study design	Qualitative	64	66.7
	Quantitative	11	11.5
	Mixed methods	11	11.5
	Reviews	10	10.4
Total		96	100%
Population (number of studies)	Patients	30	31.3%
	Caregivers	6	6.3%
	HCPs	27	28.1%
	Multiple groups	33	34.4%
Total		96	100%
Sample (number of participants	Patients	6066	53.3%
	Caregivers	3007	26.4%
	HCPs	2311	20.3%
Total		11384	100%
Phase in the trajectory	Acute	22	22.9%
	Follow-up	41**	42.7%
	Mixed	33	34.4%
Total		96	100%
Follow-up phases (N=74)	Inpatient rehabilitation	40	54.1%
	Outpatient care	1	1.4%
	Transition from hospital to home	8	10.8%
	Multiple	25	33.8%
Total		74**	100%

* International collaborations were counted separately for each country affiliation

** The total of follow-up phases (74) exceeds follow-up studies (41) as some studies included multiple rehabilitation settings

### Operationalisation of person-centred practice

Out of 96 included studies, 66 (69%) did not report using a theoretical framework or model, relying instead on broad concepts such as patient engagement, shared decision-making, or person‐centred care. The remaining 30 studies (31%) explicitly employed a recognised framework or theory. Frequently cited frameworks and theories included the International Classification of Functioning, Disability and Health, social cognition theory, normalisation process theory, and PCP frameworks.

**Fig. 2:**
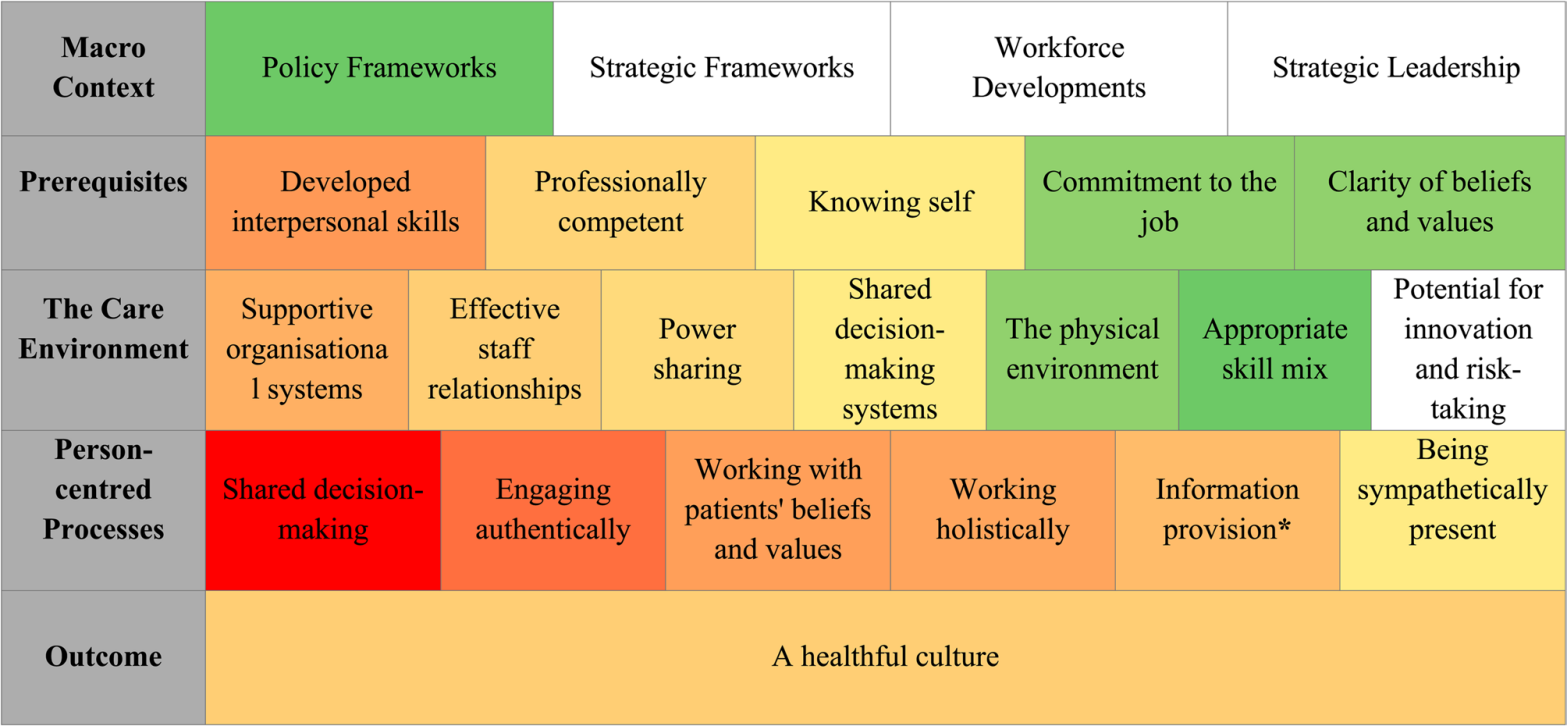
Operationalisation of person-centred practice in the stroke patient trajectory *Emerged from the data and not a construct of the framework

Figure 2 illustrates PCP components covered in the included studies versus those that remain underexplored, using a heatmap to visualise their frequency. Red, orange, yellow, green, and white indicate the scale from frequent to infrequent. The figure indicates that ***person-centred processes*** received the highest attention compared to other levels of the PCP framework with *shared decision-making*,* engaging authentically*,* working with patients’ beliefs and values*,* and working holistically* being the most emphasised components. Staff ***prerequisites*** such as *developed interpersonal skills* and *professional competence* also appear as prominent components. The component *potential for innovation and risk‐taking* was rarely addressed in the literature. ***Outcomes*** were addressed to a limited extent, while ***macro‐context*** components were almost absent in the included literature.

### Facilitators and barriers for person-centred practice

PCP facilitators and barriers were identified across five categories (Table 3).

**Table 3:** Facilitators and barriers for person-centred practice in the stroke patient trajectory

Categories	Facilitators	Barriers
Care environment and organisation	Organisational [42, 43, 64, 75], and policy [76] support Leadership support [77] and ward readiness [77]. Designated coordination roles, activities, and services [39, 52, 57, 78–82] Implementation of care models [83] and reported outcome measures [84] Co-production [62] and champions [62, 85] Adapted and personalised technologies and tools [86–89] Balanced private/social spaces for patients and informal caregivers [59, 79, 90] Favourable workspaces for HCPs [59] Familiarity and family-friendly environments [59, 60, 79, 81, 91]	Lack of resources (time, staff, funding, evidence) [11, 12, 41, 42, 44, 50, 52, 58, 64, 75–79, 82, 88, 90–109] Organisational cultures [12, 62, 76, 79, 103, 110, 111] including paternalistic approaches [76, 90, 91, 94, 96, 104, 110, 112–114] Implementation complexity [54, 62, 76, 77, 85] Standardisation [12, 39, 60, 91, 99, 104] and restrictive policies [43, 87, 90] Lack of continuity and structure [77, 78, 85, 96, 115] Technology integration and usability issues [87] Unsuitable [43, 59, 81, 92, 93, 100] and unpredictable environments [75, 109] Privacy concerns [39, 59, 90, 91] Limited use of home environment [60]
Communication and shared decision-making	Personalised supportive conversation techniques and tools [43, 44, 54, 55, 76, 97, 98, 116] HCPs’ [42, 43, 54, 76, 90, 103, 107, 117] and community [90] support and competence Early and explicit patient engagement and autonomy [14, 40, 46, 51, 104, 115, 116] Use of shared-decision making models and tools [65, 83] Tailored, phased, and repeated information [14, 39, 44, 48, 65, 83, 94, 104, 116, 118–120] Use of various information provision methods, tools, and aids [79, 89, 115, 118] Use of standardised [44, 50, 100, 106, 121] yet flexible [53, 58] instruments for goal-setting Face-to face shared-goal setting [50, 120, 122] and assessment [57] Team goals derived from patient/informal caregivers goals [11, 123] Meaningful goals and activities [50, 104] Breaking down goals for the patient [103]	Stroke related impairments, severity, and urgency [10, 42, 45, 57, 76, 77, 82, 90, 95, 97, 98, 100, 101, 107, 116, 119] Communication gaps and bypasses [45, 46, 51, 63, 79, 86, 109, 110, 113, 124] Information gaps and mismatch [44, 45, 47, 86, 91, 113–115, 119, 125, 126] Lack of knowledge and skills [43, 76, 90, 97, 98, 101] Differences in perspectives, disagreements, and uncertainty [11, 63, 100] The acute setting [10, 63, 116] Focus on recovery instead of adaptation [41, 95, 100, 127] Difficulties in communicating quality of life impact [118] Tools complexity [77] Limited options for alternative treatments [79] Complexity of interventions [118, 119] Conservative estimation of outcomes [11] Goals misalignments [11, 12, 44, 95, 100, 111] Lack of meaningful goals [81] Patient’s passive attitude [122] Complexity of fitting patient’s priorities into measurable goals [122]
Patient participation	Consideration for patient’s vulnerability, while maintaining respect and dignity [43, 44, 51, 78, 80, 91, 101, 107, 128] Building trust [42, 53, 66, 79, 104, 112, 120, 129] Meaningful therapy [105, 128], proactivity [44, 64, 103, 120, 129, 130] Self-efficacy, and motivation strategies [51, 64, 66, 91, 100, 105, 114, 131] One-on-one interactions[53] and time to listen [44, 101, 107] Awareness of patients’ perspectives, needs, and life roles [11, 39, 65, 102, 111] Meeting at the right time and warm interactions [101] Scheduled and predictable meetings [65] Holistic care through home-based services [75] Cultural and spiritual sensitivity [47, 124] peer support systems [96]	Physical, psychological, and emotional impact of stroke [39, 42, 45, 47, 50, 51, 57, 66, 76, 77, 85, 90, 91, 100, 103, 106, 107, 113, 116, 118, 127, 132] Lack of support [44, 51, 57, 60, 80, 94, 102, 112, 115, 122] Dysfunctional therapeutic relationship [12, 42], paternalistic and overprotective approaches from informal caregivers and HCPs [39, 44, 51, 77, 91, 104, 120, 124] Inequalities and lack of health literacy and education [48, 50, 79, 100, 104] Passive attitudes, vulnerability, and confidence issues [44, 64, 79, 91, 128] Lack of holistic approach and consistency [11, 12, 42, 46, 47, 50, 57, 78, 99, 102, 109] Lack of familiarity and meaning [39, 52, 91, 104] Culture and linguistic differences [100, 110] False hope delimiting peer support [96]
Interprofessional practice	Regular multidisciplinary rounds and meetings [121] HCPs’ autonomy, adaptability, and self-awareness [64, 75, 101] Team climate, expertise recognition, and skills transfer [75] Peer support and observations [54, 82, 83, 85] Shared commitment for holistic care and quality improvement [42, 64, 82] Interprofessional training [62, 76, 85, 87]and reflection [91, 111]	HCPs personality and behaviour [56, 92] Professional role perception and gatekeeping [57, 62, 92] Limited staff involvement [69, 78, 82] Lack of respect for patient’s communication abilities [43] Professional vulnerability and confidence issues [107, 109, 111] Lack of stroke specific knowledge and training [76, 82, 85, 109] Resistance to peer observations [85]
Caregivers as partners in care	Early outreach [47, 91, 126] and inclusion in decision-making [39, 47, 52, 53, 79, 101, 112, 116, 126, 128, 133] Two way communication [112] Personalised information and education [48, 49, 52, 91] Informal caregivers’ motivation [44, 49, 86, 131], support, and advocacy [63, 81, 91, 119, 133] Flexible visiting policies [125], weekend passes [79]	Informal caregivers’ disempowering and exclusion [52, 63] Informal caregivers’ other commitments and demands [49, 127] Overwhelming demands [126] Lack of processes for involvement [52, 126] and support [126] De-prioritisation in acute care [101]

Facilitators related to *the care environment and organisation* focused on organisational readiness, supportive leadership, and clear structures such as designated coordination roles, co-production, and “champions.” Barriers included limited resources, paternalistic cultures, unsupportive policies, and the complexity of implementing new care models and technologies.

*Communication strategies and shared decision-making* facilitators emphasised the importance of personalised and repeated information, tools for engaging patients in setting goals, and early promotion of patient autonomy. Barriers included insufficient knowledge and skills, communication gaps, stroke severity, and mismatched goals and expectations.

*Patient participation* facilitators highlighted respect for vulnerability and dignity, building trust, and providing motivational support through self-efficacy strategies. Barriers included paternalistic attitudes, low health literacy, inconsistent holistic care partnerships, and passive patient attitudes.

*Interprofessional practice* facilitators included regular multidisciplinary collaboration, team adaptability, and peer observation or reflection activities. Barriers included gatekeeping behaviours, variable staff involvement, and insufficient training.

*Caregivers as partners in care* was supported by early outreach, two-way communication, tailored education, and policies that enabled active involvement and advocacy. Barriers included a lack of involvement structures, overwhelming demands, and deprioritisation in certain settings.

Facilitators and barriers related to the phase of care were analysed across both the acute and follow-up phases. Several elements were common to both, but some were more specific to or prominent in one phase. In the acute phase, consistent facilitators included repeated, phased, and tailored information, effective risk communication, empathy, and the importance of caregivers acting as proxies. In the follow-up phase, consistent facilitators included healthcare professionals’ skills and strategies, tailored information and communication, shared goal setting, and continuity of care. Regarding phase-related barriers, the acute phase was marked by time pressure, stroke-related effects and complexity, information gaps, and limited resources. In the follow-up phase, consistent barriers included goal mismatches, paternalism, lack of information and education, lack of continuity in care, standardisation, and insufficient resources and training.

### Recommendations for person-centred practice

Table 4 represents recommendations under each identified category to guide implementation, practice, and assessment. *Organisation and care coordination* encompass elements related to guidelines for informed consent, privacy, and participation, developing stroke pathways with a focus on transitions, person-centred interventions, and user input.

**Table 4:** Recommendations for person-centred practice in the stroke patient trajectory

Categories	Recommendations
Organisation and care coordination	Develop guidelines for informed consent, privacy, and participation [10, 39, 76, 90, 91, 108, 114, 134] Develop person-centred interventions and strategies [47, 54, 61, 64, 66, 94] Balance different types of evidence adapted to local contexts and user inputs [50, 77, 135] Development and improvement of stroke pathways focusing on transitions [83, 85, 94, 109] Structures for organisational commitment for change [64, 82], clear documentation [127], and emotional support for HCPs [42]
Patient participation and holistic approach	Ensure integration of patient perspectives and close collaboration [11, 40, 78, 79, 85, 91, 102, 120, 127, 133, 136] Consider psychological, emotional, cultural, and social needs beyond functional recovery [46, 47, 51, 54, 94, 99, 101, 103, 109, 132] Promote equality, respect, and trust in care relationships [45, 64, 107] Knowledge assessment and providing individualised and timely education for patients [44, 48, 79, 80, 100, 120, 132, 137] Support for self-determination, self-management skills and autonomy [39, 62, 63, 90, 91, 120, 132] Ensure flexibility and ease of participation including structured and supervised peer-support [61, 96, 138, 139] Establishing meaningful goals, linked to treatment plans and [46, 127] incorporating outcome measures [84]
Caregivers’ involvement	Establish routines for involvement in patient’s needs assessment, goal setting, and rehabilitation process [11, 52, 85, 91, 115, 125, 127] Create support systems enabling information, training, and education [52, 63, 91, 100, 132, 137] Promote a proactive interdisciplinary attitude towards caregivers’ engagement [63, 112] Establish a standardised update post-procedures considering caregivers’ emotional state in the acute care [10, 63] Recognise and support informal caregivers’ advocacy and support for the patient. [90, 133]
Teamwork	Organise for regular multidisciplinary team ward rounds to improve patient needs assessment and patient involvement [44, 121] Involve all relevant HCPs in rehabilitation teams [52, 78, 79, 137] Involve speech language pathologists as consultants in communication support [76] Ensure HCPs engagement in deliberation, evaluation, and reflexion [91] Implement training on clinical skills, communication [39, 43, 53, 76, 82, 90, 97, 112, 118, 122, 129, 133], shared decision making [62, 82, 85, 103], person-centred practices [12, 54, 134, 139], and stroke-specific education [10, 109] Promote knowledge on vulnerable patients, motivational strategies [105] and emotional support for stroke survivors and their caregivers [101, 110, 112] Promote skills transfer, coaching abilities, and work-based learning and development [82, 111]
Communication	Develop systems and policies that acknowledge and address patients’ communication needs [43, 54, 76, 97, 109, 113, 119] Provide appropriate training for HCPs and volunteers on communication strategies [42, 76, 82, 90, 97, 98, 107] Provide better communication about status and progress, ensuring communication plan updates [45, 54, 76] Promote respectful two-way communication [52, 79] Allocate enough time for patients with aphasia [42, 107]
Shared decision making	Establish shared decision-making processes [45, 57, 60, 76, 100, 108, 116] Establish decision making capacity assessment strategies, and provide risk/benefit information [116, 119] Invite patients and caregivers to participate in clinical reasoning [9] Establish contextual and flexible goals and a shared understanding of goals, practiced through the multidisciplinary team[100, 103] Provie clinicians with pragmatic tools that can be integrated into practice [106] Manualising the documentation of person-centred goal setting [58]
Information provision	Provide multimodal and precise information tailored to individual needs [10, 45, 51, 63, 91, 116] Spread information strategically throughout hospital stay adapting it to changing needs [79, 80, 109, 115, 119] Invest resources on information provision, assisting HCPs in producing better information [10, 134] Standardise risk/benefit information built on evidence-based data, and guidelines on information provision and delivery [115, 118] Develop information materials based on evidence-based practice information quality criteria (EBPI) [139] Provide an overview of available care and services options [134]
Physical environment	Create evidence-based unit designs integrating HCP and user inputs [43, 59] Integrate environment as a resource in rehabilitation processes [60, 100] Balance private and social spaces to prevent isolation, while adjusting care routines to single rooms under monitoring [59, 90] Create flexible environment allowing patients exploration and autonomy [79, 90] Create an inclusive environment for informal caregivers [52]
Tools	Develop and implement digital solutions with user inputs ensuring usability [86–88, 132] Develop decision capacity assessment and shared decision-making tools [109, 118] Develop and implement tools for biopsychosocial needs assessment [137], discharge planning checklists [76], and supportive conversation [82] Better incorporation of tools and techniques to support the involvement of all severity groups [95] Reconsider the reliance on SMART goals [106]

*Patient participation and holistic approach* emphasise close collaboration incorporating all patient needs beyond functional recovery, with individualised and timely information and education being critical.

*Caregivers’ involvement* stresses the need to establish involvement routines, support systems, and a proactive, positive attitude from the multidisciplinary team.

*Teamwork* includes healthcare professionals’ stroke knowledge and skills development through training, deliberation, and reflection.

*Communication* entails understanding the communication needs of patients with stroke. It is important to train healthcare professionals and volunteers on communication strategies and ensure that patients with aphasia are provided with enough time for interactions.

*Shared decision making* requires patient and caregiver participation in clinical reasoning and providing HCPs with pragmatic tools for goal setting.

*Information provision* must be multimodal, precise, and tailored to patients’ needs, distributed strategically throughout the patient trajectory and not concentrated during transitions.

*Physical environment* should support stroke care and rehabilitation by balancing private and social spaces, with flexibility and inclusivity as important characteristics.

*Tools* include digital solutions for supportive conversations, shared decision-making, capacity assessment, as well as for supporting transitions.

### Evolution of research gaps in person-centred practice

**Fig. 3:**
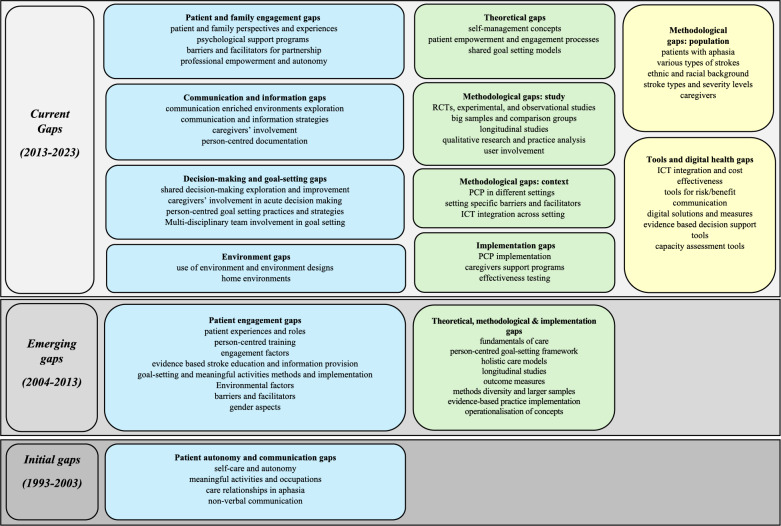
Research gaps for person-centred practice in the stroke patient trajectory Description: Person-centred practice gaps are blue, theoretical, methodological, and implementation gaps are green, while research gaps that emerged over the last decade are yellow

Figure 3 summarises research gaps over the past three decades providing insights into the evolution of PCP in the stroke patient trajectory as well as highlighting persisting and current research gaps.

### 1993–2002

The early PCP landscape was characterised by a limited number of studies with a fragmented and diverse focus. Key themes that emerged from studies included autonomy [39, 40], meaningful care [41], and care relationships in aphasia and non-verbal communication [42].

### 2003–2012

Building on previous foundational studies, this period revealed a deeper understanding of PCP, touching upon patient engagement elements [43–45] and stressing the need for theoretical foundations [46, 47], rigorous methodologies [48, 49], and implementation and operationalisation methods [50, 51].

### 2013–2023

The last decade has seen a substantial number of research studies compared to the previous decades (Table 2), resulting in research gaps classified into three main categories:

1. PCP gaps, including gaps related to patient and family engagement [52, 53], communication and information [54–56], decision-making and goal setting [57, 58], and environmental gaps [59, 60].
2. Theoretical, methodological, and implementation gaps, including several persisting gaps from previous decades [54, 61–64].
3. Tools and digital health gaps, emphasising the need for digital solutions and tools supporting PCP [65, 66].

## Discussion

This scoping review collected a wide variety of studies on PCP in the stroke patient trajectory, spanning the acute and follow-up phases, various study designs, and involving patients, healthcare professionals, and caregivers. However, little or no research was found on prehospital care, outpatient rehabilitation, and transitional care. Within PCP, the components of shared decision-making, engagement, and interpersonal skills dominate the literature, while research in the macro context is lacking.

Over the past three decades, research on PCP in the stroke patient trajectory has grown considerably, paralleling emerging PCP research and theory [67, 68], and a global policy shift [18]. Early work focused mainly on patient autonomy and communication; however, more recent studies capture the broader scope of PCP, with a focus on operational aspects of PCP, such as shared goal-setting and information provision. More than half of the studies originated from Australia, Sweden, the UK, and the Netherlands, likely reflecting the presence of PCP institutions in these countries that lead research and framework development. Most studies originated from high-income countries, resulting in a gap in clinical and cultural insights specific to middle- and low-income countries. Qualitative designs predominated, exploring experiences and perspectives, while fewer quantitative and mixed-methods studies addressed implementation or outcomes.

Two-thirds of the included studies did not reference a theoretical framework. Nonetheless, all could be mapped to the PCP framework [27], suggesting that it captures the diverse aspects studied in the stroke patient trajectory. Even so, the mapping showed an imbalanced coverage of the framework components in the literature. Information provision consistently emerged from the literature as an important element in stroke care. Although not an explicit component of the PCP framework, it is fundamentally embedded in other elements of the framework, such as engaging authentically and shared decision-making, both of which rely on effective communication and information. This emphasis on information across studies raises the question of whether communication and information provision should be more explicitly represented in the PCP framework. Future studies would benefit from exploring various theoretical foundations—including, but not limited to, the PCP framework—to enhance conceptual clarity and improve the implementation of PCP in the stroke patient trajectory.

Our results show that PCP faces both similar and distinct barriers across stroke phases [69, 70]. Time, urgency, and the patient’s situation are particularly important in the acute phase, while other barriers emerge in the follow-up phase. A literature review exploring PCP in emergency departments showed that the environment impacts how staff engage in care [71] and noted that PCP had not been explored in the emergency room setting. In our analysis, communication and information provision appeared as dominant components in the acute phase. However, more aspects of PCP emerged in follow-up and rehabilitation phases such as goal mismatches, lack of continuity, and standardisation. Patients with stroke described their experience during the trajectory as a transformative journey, moving “from a recipient to an active contributor to their own care” [72]. Mapping differences in PCP across phases is important yet challenging, primarily due to the lack of clear definitions for these phases in the included articles.

Across multiple studies, organisation-related facilitators and barriers were emphasised. Strong policy support and designated coordination roles were essential facilitators, echoing the WHO framework on integrated, people-centred health services [18]. Conversely, lack of resources and paternalistic approaches were mentioned by a substantial number of studies. Similar barriers have been identified in other acute hospital settings, where the busy and stressful nature of the acute setting is in itself a barrier to PCP [28].

Key facilitators for communication and shared decision-making included supportive conversation techniques, tailored and phased information, shared decision-making models and tools, as well as healthcare professionals’ support and competence. Joseph-Williams et al. identified knowledge imbalance and power dynamics as important barriers to shared decision-making, emphasising the importance of information provision and education [73]. The emphasis on information provision in the included articles is also supported by research showing improved outcomes related to tailored information provision [74]. Overall, successful communication and shared decision-making require not only practical translation but also interventions at multiple levels: organisational, professional, and strategic. Stroke-related physical, psychological, and cognitive impairments add further complexity, often reinforcing paternalistic practices.

The PCP framework helps illustrate how our identified facilitators, barriers, and recommendations are interconnected. For example, informal caregivers’ involvement emerged as a facilitator for both communication and patient participation. This interconnection shows how the components of PCP function as an integrated framework rather than isolated components. Achieving PCP thus requires addressing multi-dimensional barriers while prioritising relationship building.

There are limitations to this study that need mentioning: the scoping review is focused on the three months following stroke onset (the stroke patient trajectory), which limits the number of studies included. The argument for this choice is to have a focus on the acute and sub-acute phases as there is more extensive knowledge in the chronic phase of stroke. Only 10% of the study screening was done independently with blinding enabled by two reviewers, potentially reducing the reliability, though mitigated by team discussions. The deductive analysis of PCP components may have introduced subjectivity. Limiting the review to English-language studies could have led to the exclusion of relevant evidence. Additionally, the quality of included studies was not assessed.

## Conclusions

This scoping review of PCP in the stroke patient trajectory revealed several key findings. First, the focus in the literature is on person-centred processes, specifically shared decision-making and engaging authentically. Second, facilitators and barriers emerged across several categories, highlighting the broad nature of PCP. Recommendations for practice covered both organisational and relational aspects.

Several research gaps were identified in the review, including a limited representation of core PCP components beyond shared decision-making and authentic engagement, alongside theoretical, methodological, and implementation gaps. There is a need for more robust study designs, including RCTs, larger sample sizes, longitudinal studies, and qualitative research to better understand the broader PCP components. Further research is therefore needed to explore underrepresented aspects of PCP and to develop more targeted interventions and implementation strategies.

## Supplementary Information

Supplementary Material 1

## Data Availability

The datasets supporting the conclusions of this article are included within the article and in Additional file 1. The scoping review protocol is available at: https://osf.io/wyd8e.

## Appendices

### Appendix I: Search strategy for CINAHL

Interface - EBSCOhost Research Databases

Search Screen - Advanced Search

Database - CINAHL with Full Text

**Table:**

**#**	**Query**	**Limiters/** **Expanders**	**Results**
S5	S3 AND S4	Search modes - Boolean/Phrase	1,064
S4	S1 AND S2	Search modes - Boolean/Phrase	2,456
S3	(MH "hospital") OR (MH "patient trajectory") OR (MH "care pathway") OR (MH "in-hospital") OR (MH "rehabilitation") OR (MH "pre-hospital") OR (TI "hospital") OR (TI "patient trajectory") OR (TI care pathway*) OR (TI "in-hospital") OR (TI "rehabilitation") OR (TI "Prehospital") OR (TI "acute care") OR (TI "emergency room") OR (TI "emergency department") OR (TI "ambulance") OR (TI "stroke unit") OR (TI "stroke units") OR (TI "care transition") OR (TI "nursing home*") OR (TI "outpatient") OR (TI "home care") OR (TI "community care") OR (TI "recovery") OR (TI "home visit") OR (TI "home visits") OR (TI "post-hospital") OR (TI "day care") OR (TI "inpatient treatment") OR (TI "inpatient care") OR (TI "emergency ward") OR (TI "ambulatory care") OR (TI "care pathways") OR (TI "care trajectories") OR (TI "hospitalization") OR (AB "hospital") OR (AB "patient trajectory") OR (AB care pathway*) OR (AB "in-hospital") OR (AB "rehabilitation") OR (AB "Prehospital") OR (AB "acute care") OR (AB "emergency room") OR (AB "emergency department") OR (AB "ambulance") OR (AB "stroke unit") OR (AB "stroke units") OR (AB "care transition") OR (AB "outpatient") OR (AB "home care") OR (AB "community care") OR (AB "recovery") OR (AB "home visit") OR (AB "home visits") OR (AB "goal setting") OR (AB "post-hospital") OR (AB "nursing home*") OR (AB "day care") OR (AB "inpatient treatment") OR (AB "inpatient care") OR (AB "emergency ward") OR (AB "ambulatory care") OR (AB "care pathways") OR (AB "care trajectories") OR (AB "hospitalization") OR (AB "care")	Search modes - Boolean/Phrase	768,399
S2	((MH "Stroke") OR (MH "Stroke Patients")) OR (TI(cva OR cvas OR poststroke* OR stroke* OR apoplex*) OR AB(cva OR cvas OR poststroke* OR stroke* OR apoplex*)) OR ((TI(brain* OR cerebr* OR cerebell* OR intracran* OR intracerebral* OR vertebrobasilar*) OR AB(brain* OR cerebr* OR cerebell* OR intracran* OR intracerebral* OR vertebrobasilar*)) N3 (TI(vascular*) OR AB(vascular*)) N3 (TI(disease OR diseases OR accident* OR disorder*) OR AB(disease OR diseases OR accident* OR disorder*))) OR (TI(cerebrovascular*) OR AB(cerebrovascular*) N3 (TI(disease OR diseases OR accident* OR disorder*) OR AB(disease OR diseases OR accident* OR disorder*))) OR ((TI(brain* OR cerebr* OR cerebell* OR intracran* OR intracerebral* OR vertebrobasilar*) OR AB(brain* OR cerebr* OR cerebell* OR intracran* OR intracerebral* OR vertebrobasilar*)) N3 (TI(haemorrhag* OR hemorrhag* OR ischemi* OR ischaemi* OR infarct* OR haematoma* OR hematoma* OR bleed*) OR AB(haemorrhag* OR hemorrhag* OR ischemi* OR ischaemi* OR infarct* OR haematoma* OR hematoma* OR bleed*))) OR (DE "Hemiplegia" OR DE "General Paresis" OR TI(hemipleg* OR hemipar* OR paresis OR paretic) OR AB(hemipleg* OR hemipar* OR paresis OR paretic)))	Search modes - Boolean/Phrase	162,688
S1	(MH "Decision Making") OR (MH "Consumer Participation") OR (MH "Professional-Patient Relations") OR (MH "Physician-Patient Relations") OR (MH "Nurse-Patient Relations") OR (MH "Patient Centered Care") OR TI "Shared decision") OR (TI "Shared decisions") OR (TI "Patient participation") OR (TI "consumer participation") OR (TI "Participating patient") OR (TI "Participating patients") OR (TI "patient contribution") OR (TI "patients contribution") OR (TI "patient contributions") OR (TI "patients contributions") OR (TI "Patient involvement") OR (TI "Physician-Patient Relation") OR (TI "Nurse-Patient Relation") OR (TI "Physician-patient communication") OR (TI "Patient-focused care") OR (TI "Patient-focused intervention") OR (TI "Patient-centered") OR (TI "Patient-centred") OR (TI "patient centredness") OR (TI "Individualized-care") OR (TI "person-centered care") OR (TI "person-centred care") OR (TI "client-centered care") OR (TI "client-centred care") OR (TI "resident-centered care") OR (TI "resident-centred care") OR (TI "patient empowerment") OR (TI "Patient Information") OR (TI "patient communication") OR (AB "Shared decision") OR (AB "Shared decisions") OR (AB "Patient participation") OR (AB "consumer participation") OR (AB "Patient interviews") OR (AB "Participating patient") OR (AB "Participating patients") OR (AB "patient contribution") OR (AB "patients contribution") OR (AB "patient contributions") OR (AB "patients contributions") OR (AB "Patient involvement") OR (AB "Physician-Patient Relation") OR (AB "Physician-Patient Relations") OR (AB "Physician-patient communication") OR (AB "Patient-focused care") OR (AB "Patient-focused intervention") OR (AB "Patient-centered") OR (AB "Patient-centred") OR (AB "patient centredness") OR (AB "Individualized-care") OR (AB "person-centered care") OR (AB "person-centred care") OR (AB "client-centered care") OR (AB "client-centred care") OR (AB "resident-centered care") OR (AB "resident-centred care") OR (AB "patient empowerment") OR (AB "Patient Information") OR (AB "patient communication") OR (AB "patient activation") OR (AB "patient engagement") OR (TI " family centered care") OR (TI " family centred care") OR (TI " family involvement") OR (TI "kin involvement") OR (TI " relative involvement") OR (AB " family centered care") OR (AB " family centred care") OR (AB " family involvement") OR (AB "kin involvement") OR (AB " relative involvement")	Search modes - Boolean/Phrase	236,058

### Appendix II: Data extraction instrument

**Table:**

Article details (authors, year, and country)
Aim
Key findings
Who participated in the study (patient, informal caregiver, HCPs)
Sample size
Study design
Stroke care setting (Acute, Follow-up, Mixed)
Underlying theories
PCP components
Facilitators for PCP in the stroke patient trajectory
Barriers for PCP in the stroke patient trajectory
Recommendation for practice
Research Gaps

## Abbreviations

PCP: Person-centred practice
HCPs: Healthcare professionals
